# Metabolomics analysis identifies metabolites associated with systemic acquired resistance in Arabidopsis

**DOI:** 10.7717/peerj.10047

**Published:** 2020-09-30

**Authors:** Hang Gao, Qian Zhou, Liu Yang, Kaili Zhang, Yeye Ma, Zi-Qin Xu

**Affiliations:** 1 Key Laboratory of Resource Biology and Biotechnology in Western China (Ministry of Education), Shaanxi Provincial Key Laboratory of Biotechnology, College of Life Sciences, Northwest University, Xi’an, Shaanxi, People’s Republic of China; 2 Shanghai Omicsspace Biotechnology Co. Ltd., Shanghai, People’s Republic of China

**Keywords:** Metabolomics, Systemic acquired resistance, Arabidopsis, Amino acids, Phenolic compounds

## Abstract

**Background:**

Systemic acquired resistance (SAR) is a type of plant defense response that provides a long-lasting resistance to broad-spectrum pathogens in uninfected distal tissues following an initial localized infection. However, little information is available at present on the biological basis of SAR at the molecular level, especially in uninfected distal leaves.

**Methods:**

In the present work, we used two SAR-inducing pathogens, avirulent *Pseudomonas syringae* pv. *maculicola* ES4326 harboring *avrRpm1* (*Psm avrRpm1*) and virulent *P. syringae* pv. *maculicola* ES4326 (*Psm* ES4326), to induce SAR in Arabidopsis ecotype Col-0. A metabolomics approach based on ultra-high-performance liquid chromatography (UPLC) coupled with mass spectrometry (MS) was used to identify SAR-related metabolites in infected local leaves, and in uninfected distal leaves.

**Results:**

Differentially accumulated metabolites were distinguished by statistical analyses. The results showed that both the primary metabolism and the secondary metabolism were significantly altered in infected local leaves and in uninfected distal leaves, including phenolic compounds, amino acids, nucleotides, organic acids, and many other metabolites.

**Conclusions:**

The content of amino acids and phenolic compounds increased in uninfected distal leaves, suggesting their contribution to the establishment of SAR. In addition, 2′-hydroxy-4, 4′, 6′-trimethoxychalcone, phenylalanine, and *p*-coumaric acid were identified as potential components which may play important roles both in basic resistance and in SAR. This work provides a reference for understanding of the metabolic mechanism associated with SAR in plants, which will be useful for further investigation of the molecular basis of the systemic immunity.

## Introduction

During the long-term co-evolution with microorganisms, plants have evolved multiple strategies to antagonize the colonization by pathogens, including the constitutive and inducible defenses (Spoel & Dong, 2012). Briefly, the constitutive defenses refer primarily to pathogen-associated molecular pattern (PAMP)-triggered immunity (PTI) and effector-triggered immunity (ETI) (Zhang et al., 2018). PTI is a type of basal resistance response induced by the components with a conserved structure in pathogens, such as fungal chitin and bacterial flagellin (Nürnberger & Brunner, 2002; Kunze et al., 2004). Different from this, ETI is established by plants following the recognition of the virulent pathogenic effectors by immune receptors. ETI is usually accompanied by a hypersensitive response (HR) in the form of rapid cell death at the infection sites, which can provide effective local resistance to pathogens (Johansson et al., 2015). Although ETI is generally associated with stronger local responses than PTI, they trigger similar immunity by expressing partially overlapping genes, suggesting the synergistic effects of these genes in PTI and ETI (Bhattacharjee et al., 2011).

Besides constitutive defense, plants are also able to initiate inducible defense in tissues distant from the infected sites, namely systemic acquired resistance (SAR). As a defense response induced in systemic organs by prior localized infection, SAR confers enhanced resistance against a variety of pathogens (Cameron, Dixon & Lamb, 1994). The priming of SAR in systemic tissues is related to signals coming from local infection sites. By activation of pathogenesis-related genes, including *PR1*, *PR2*, and *PR5*, SAR can increase the capacity of the plants to cope with the imminent infection by the pathogens (Gruner et al., 2013).

Although ETI and PTI at the infection sites are believed to be necessary for establishment of the systemic resistance, some research confirms that PAMP alone is sufficient in induction of SAR (Shah & Zeier, 2013). During the initial stage of SAR, signal molecules are obviously being passed from locally infected sites to systemic tissues, which will lead to a transcriptional reprogramming in distal uninfected locations. These signal molecules include glycerol-3-phosphate (G3P), methyl salicylate (Me SA), azelaic acid (AzA), pipecolic acid (Pip), and dehydroabietinal (DA) (Park et al., 2007; Jung et al., 2009; Chanda et al., 2011; Gruner et al., 2013; Zhang et al., 2018). It has been found that the accumulation of pipecolic acid (Pip) in local and systemic tissues is crucial for the induction of SAR (Návarová et al., 2012; Hartmann et al., 2018). N-hydroxypipecolic acid (NHP), a direct derivate of Pip, has been confirmed to be a pivotal inducer of SAR (Hartmann et al., 2018; Chen et al., 2018).

Systemic responses to long distance signals have been studied at the physiological, transcriptional and metabolomic levels (Gruner et al., 2013; Schwachtje et al., 2018). Except for the small molecules, several proteins have been confirmed to be related to signaling of systemic immunity (Chanda et al., 2011; Carella et al., 2016). For example, the long distance movement of DEFECTIVE IN INDUCED RESISTANCE1 (DIR1) from infected leaves to systemic leaves through plasmodesmata is crucial to the establishment of SAR (Chanda et al., 2011). During the triggering process of SAR, the genes associated with salicylic acid (SA)-defense pathway were induced, whereas the genes associated with jasmonate (JA)/ethylene (ET)-defense pathways and cell wall remodeling were repressed in systemic non-inoculated tissues.

The primary and the secondary compounds related to defense mechanisms can be analyzed simultaneously by metabolite profiling methods (De Vos et al., 2007). Generally, metabolomics involves both the qualitative and quantitative analyses of all the metabolites in organisms (Dettmer, Aronov & Hammock, 2007). Plants can produce a large variety of compounds with a wide range of chemical diversity and content (De Vos et al., 2007). To identify as many metabolites as possible in a single step, a suitable method for sample analysis is required. Based on the high resolution and the high mass accuracy, UPLC-MS/MS [ultra-high-performance liquid chromatography (UPLC) and mass spectrometry (MS)] has become a typical approach in metabolomics (Schauer et al., 2006; Ahmad et al., 2011; Cao et al., 2016; Wu et al., 2018). By UPLC, the resolution, the peak efficiency, and the separation speed for complex matrices can be improved (Grata et al., 2008).

In recent years, numerous studies concerning plant metabolic changes in response to pathogens have been conducted, such as Wheat/Streak (Farahbakhsh et al., 2019), *Arabidopsis thaliana*/*Pseudomonas syringae* (Ward et al., 2010), rice/*brown planthopper* (Kang et al., 2019), and soybean/*Fusarium tucumaniae* (Scandiani et al., 2015). However, there is a limited number of studies using a metabolomics approach to elucidate the mechanism of SAR. By GC-MS (gas chromatography–mass spectrometry), Schwachtje et al. (2018) found the suppressed nitrogen metabolites and the increased organic acids contributed to SAR in systemic tissues. By NMR (nuclear magnetic resonance), Wang et al. (2016) found sugar signaling plays an important role in SAR. In the present work, we used UPLC-MS/MS to identify the metabolites associated with SAR in Arabidopsis. To this end, avirulent *P. syringae* pv. *maculicola (Psm) avrRpm1* (*Psm avrRpm1*) and virulent *Psm ES4326* were used to induce SAR in Arabidopsis, and UPLC-MS/MS was used to identify the different metabolites in infected leaves and uninfected distal leaves. The results of this study can provide comprehensive details of key metabolites associated with SAR, which is useful for further investigating the molecular basis of systemic immunity.

## Materials & Methods

### Plant materials and growth conditions

*Arabidopsis thaliana* (L.) Heynh. ecotype Col-0 plants were grown in matrix of perlite, vermiculite, and nutrient soil (1:1:1, v/v/v) in a controlled environment chamber under the conditions of 65% relative humidity (RH) and 16-h light/8-h dark cycles. Growth temperatures during the day and the night were set to 22 °C and 18 °C, respectively. Four to five-week-old plants exhibiting a uniform appearance were used as materials.

### SAR analyses

Avirulent *Psm avrRpm1* (termed as Avr) and virulent *Psm ES4326* (termed as Vir) were used for induction of SAR. Bacterium strains were cultivated at 200 r/m and 28 °C in King’s B medium supplemented with 50 mg/L streptomycin and 50 mg/L tetracycline for Avr, or 50 mg/L streptomycin for Vir, respectively. For SAR induction, a 50 µL aliquot of Avr or Vir bacterial suspension in 10 mmol/L MgCl_2_ (OD_600_ = 0.002) was infiltrated into three local rosette leaves (LL, typically leaf 7–9). 10 mmol/L MgCl_2_ was served as mock treatment (CK). 48 h later, Avr, Vir or CK inoculated local rosette leaves (termed as LL-Avr, LL-Vir or LL-CK) and distal rosette leaves (typically leaf 10–12) (termed as DL-Avr, DL-Vir or DL-CK) were harvested and stored in liquid nitrogen. Taken together, six biologically independent SAR experiments for each treatment were performed in metabolomics analysis.

### Metabolite extraction

Each sample (50 mg) was first ground in liquid nitrogen, followed by addition of 0.5 ml pre-cooled methanol/water (3:1, v/v). After vortexing, the samples were incubated for 1 h at −20 °C for protein precipitation. Then the samples were centrifuged at 13,000 r/m and 4 °C for 15 min. After filtering with a 0.22 µm filter, the supernatant (about 0.45 mL) was dried with nitrogen and stored at −80 °C. QC sample mixed with equal amount of each sample was prepared according to above steps.

### LC/MSMS analysis

The samples were redissolved with 50 µL pre-cooled mixture of methanol, isopropanol, and water (1:1:2, v/v/v), and were added into a 4 °C autosampler and separated on a DIONEX UltiMate_3000 UHPLC system using a C18 column. The volume of the sample was 3 µL, the column temperature was 45 °C, and the flow rate was 0.35 mL/min. Chromatographic mobile phase A: 0.1% formic acid; B: acetonitrile in 0.1% formic acid. Chromatographic gradient elution programme: 0–0.5min, 98% A; 0.5–15 min, A with a linear change from 98% to 2%; 15–17 min, 2% A; 17.1–20 min, A with a linear change from 2% to 98%. QC samples were inserted every 6 samples to evaluate the stability of the system and the reliability of the data.

The samples were separated by UPLC and analyzed by a Q-Exactive mass spectrometer (Thermo Scientific, San Jose, CA, USA). Each sample was electrospray-ionized (ESI) and detected with positive and negative ion mode, respectively. The ESI source conditions were as follows: spray voltage (kV), 3.5 ESI+ and 3.2 ESI- (positive and negative modes); source temperature, 320 °C; sheath gas flow rate (Arb), 45; aux gas flow rate (Arb), 15. A positive/negative data-dependent (DD) high-energy collision dissociation (HCD)-MS2 mode was used for data acquisition with full MS scan (resolution: 70,000; AGC target: 1e6; maximum IT: 100 ms; scan range: m/z 80-1200). Parameters in HCD-based data dependent MS/MS (DDMS2) acquisition: resolution, 175,00; AGC target, 5e4; maximum IT, 50 ms; loop count, 5; TopN, 5; isolation window, m/z 1.0; scan range, m/z 50–750; NCE/stepped NCE, 10, 30, 60; underfill ratio, 1.0%; intensity threshold, 8e3; dynamic exclusion, 10 s. Except for QC samples whose data were acquired using full MS scan plus DDMS2 mode, other samples were run using the full MS scan mode. Compound Discoverer 3.0 was used for search of the raw data, peak alignment, peak deconvolution, adduct treatment, compound detection, grouping, and metabolite identification. A blank sample was used for background subtraction and noise removal during the pre-processing step. The precision mass matching (5 ppm) (Cao et al., 2016), and MS^1^ and MS^2^ matching to search MZcloud and ChemSpider database were used to determine the metabolite structure. For MZcloud database, the metabolites were identified by exact mass (m/z), molecular formula, and fragmentation spectrum (MS^2^). For ChemsPider database, the metabolites were identified by exact mass (m/z) and molecular formula. The detailed workflow and the parameters are provided in Table S5. The SIMCA-P14.1 application software (Umetrics, Umea, Sweden) was used for pattern recognition. After processing by Pareto scaling, the data were subjected to multidimensional statistical analysis, including orthogonal partial least squares discriminant analysis.

### Data analysis

Pareto variance SIMCA software (Umetrics) was used to establish the orthogonal partial least squares discriminant analysis (OPLS-DA) model after data were scaled to Pareto variance (Liu et al., 2019). For analyzing differential metabolites of each comparison group, a 1.2 fold change (FC) in metabolite abundance, a VIP (variable importance for the projection) score greater than 1 and a FDR (false discovery rate) adjusted *p*-value less than or equal to 0.05 for student’s *t*-test were considered as having statistically significant differences. Hierarchical clustering of the metabolite abundance data was conducted using the Euclidean method for calculating the distance and building the linkage trees. Perseus 1.6.0.2 was used for hierarchical clustering analyses.

### RNA isolation and quantitative real-time PCR

Four-week-old wild-type Col-0 plants were used as materials in expression analysis. Avr or Vir with an OD_600_ of 0.002 were infiltrated into 3 rosette leaves. The distal uninfected leaves were used as materials in isolation of total RNA with Trizol reagent (Invitrogen). Complementary DNA (cDNA) was prepared with PrimeScript First Strand cDNA Synthesis Kit (Takara). 1.5 µL of cDNA, 5 µL FastStart Essential DNA Green Master (Roche), and gene-specific primers (0.75 µmol/L) were mixed in each PCR with a total volume of 10 µL. *ACT8* was used as a reference gene. The primer sequences were as follows: *PR1* forward: GCTCTTGTAGGTGCT CTTGTTC, *PR1* reverse: GCCTCTTAGTTGTTCTGCGTAG; *ACT8* forward: TGTGCCTATCTACGAGGGTTT, *ACT8* reverse: TTTCCCGTTCTGCTGTTGT. The amplification conditions included denaturation at 95 °C for 10 min, 39 cycles of denaturation at 95 °C for 10s and annealing at corresponding temperature for 30s. 2^−ΔΔ*Ct*^ (cycle threshold) method was used to calculate the relative expression levels. The values were normalized to those of the reference gene and to those of the mock sample [ΔΔ*Ct* = (*Ct*_*PR*1_ − *Ct*_actin_) different sample − (*Ct*_*PR*1_ − *Ct*_actin_) *CK*]. One-way repeat measures analysis of variance (ANOVA) was used to determine the significant differences.

## Results

### Phenotypes shown in *Psm*-inoculated Arabidopsis plants

**Figure 1:**
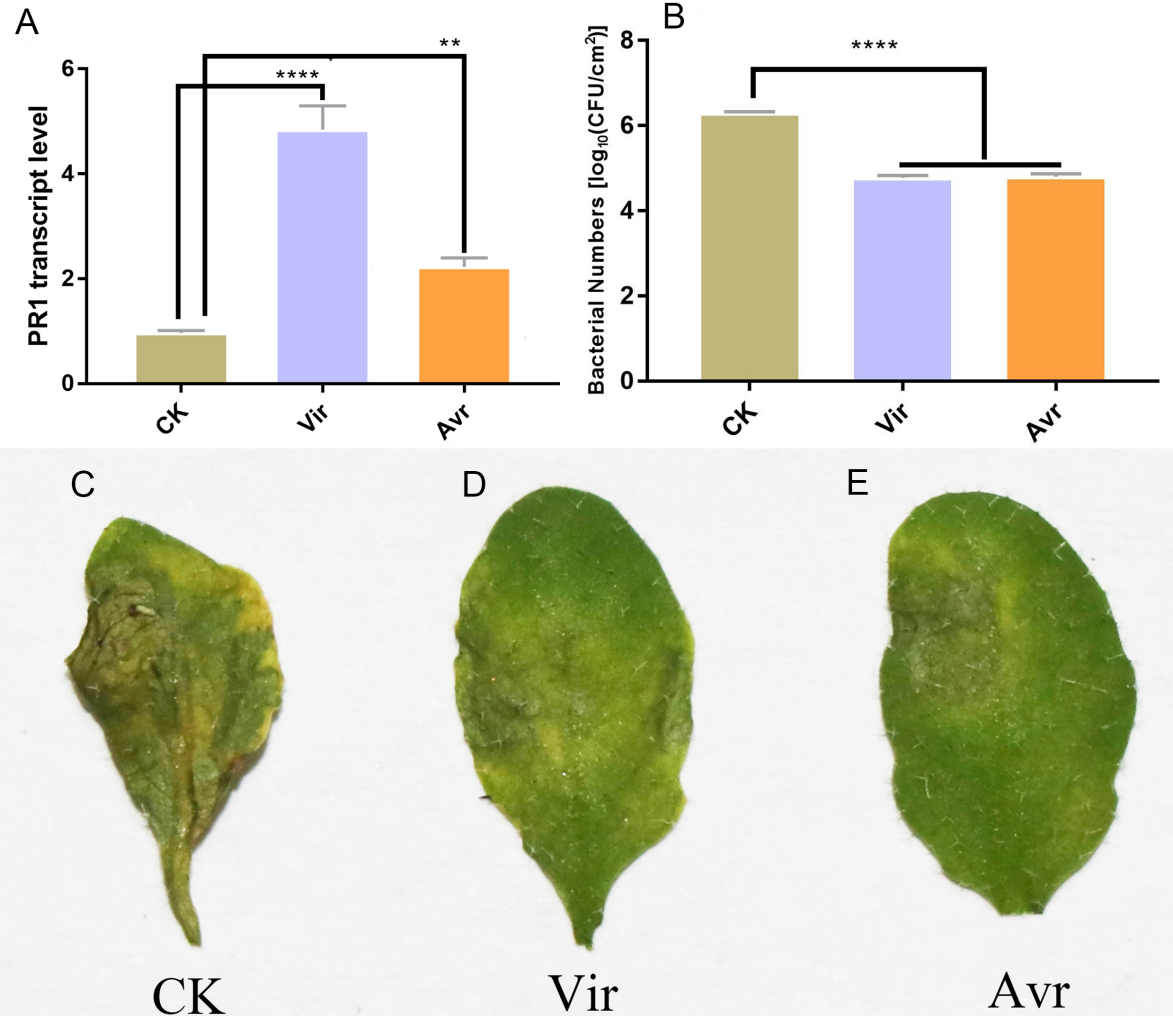
Sample evaluation of different treated groups. Arabidopsis Col-0 plants were inoculated with avirulent *Psm avrRpm1* (Avr), virulent *Psm ES4326* (Vir) and MgCl_2_ (CK). RT-qPCR and bacterial growth data represent an average of three biological replicates, One-way ANOVA was used to determine significant differences. (A) Expression analysis of PR1 gene in distal leaves (DL) of plants locally Vir and Avr inoculated. One-way ANOVA was used to determine significant differences. (B) Assessment of the bacterial growth in DL after secondary infection with Vir, when LL was infected with Avr, Vir and CK at first. ** represented *P* < 0.01, **** represented *P* < 0.0001. (C–E) Phenotype of DL after secondary infection. Local leaves was infected with Avr, Vir and CK at first, after 48 h, the DL was infected with Vir. The phenotype of DL was observed 72 h later.

Three leaves of four-week-old Arabidopsis seedlings were inoculated with SAR-inducing pathogens, avirulent *Psm avrRpm1* (Avr) and virulent *Psm ES4326* (Vir), and MgCl_2_ (CK) was used as the negative control. After 48 h, the distal leaves (termed as DL-Avr, DL-Vir, and DL-CK) were used for RT-qPCR analysis of *PR1* (AT2G14610, *PATHOGENESIS-RELATED GENE 1*, the marker gene of SAR). The result showed that the accumulation levels of *PR1* transcripts in DL-Avr and DL-Vir were higher than that in DL-CK (Fig. 1A). To evaluate the sample quality, the distal leaves of the plants locally inoculated with Avr, Vir and CK were further inoculated with Vir. 72 h later, these distal leaves secondarily inoculated with Vir were used for phenotype observation and bacteria counting. As shown in Figs. 1B and 1C–1E, DL-Avr and DL-Vir displayed a much lighter chlorosis and a less colony forming unit in comparison with DL-CK, indicating SAR was successfully induced.

To investigate the metabolomics responses of *Psm*-inoculated local leaves and uninoculated distal leaves, three local leaves of four-week-old Arabidopsis seedlings were infiltrated with Avr, Vir or CK. Two days later, the inoculated local leaves and the uninoculated distal leaves were harvested. The samples used for metabolomics analyses were divided into 6 groups, including Avr-inoculated local leaves (LL-Avr), Vir-inoculated local leaves (LL-Vir), MgCl_2_-infiltrated local leaves (LL-CK), distal leaves of the plants locally inoculated with Avr (DL-Avr), distal leaves of the plants locally inoculated with Vir (DL-Vir), distal leaves of the plants locally infiltrated with MgCl_2_ (DL-CK). Each group contains 6 biological replicates.

### Multivariate data analysis

**Figure 2:**
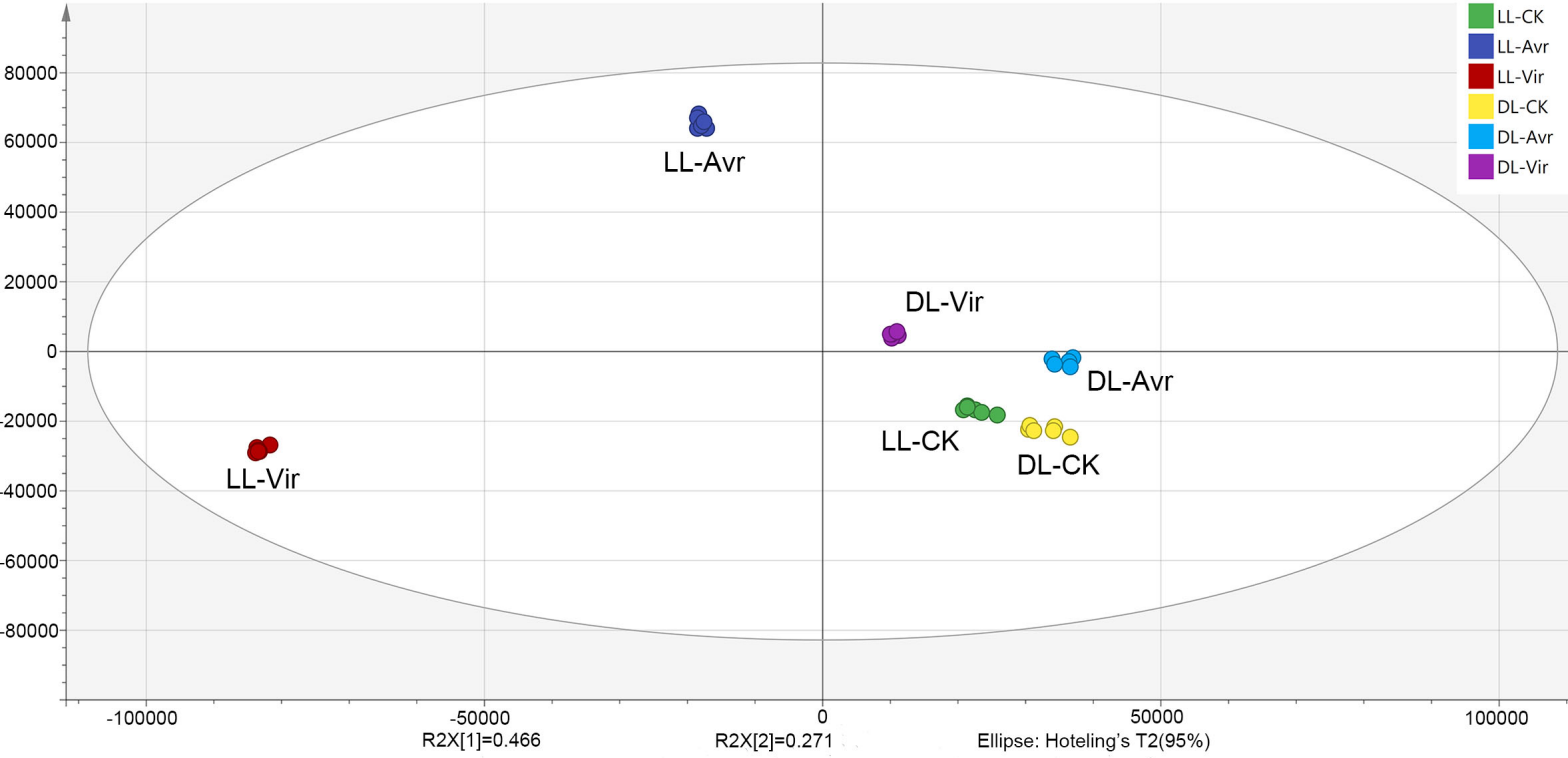
Orthogonal Partial Least Squares Discrimination Analysis (OPLS-DA) scores plots of LLAvr, LL-Vir, LL-CK, DL-Avr, DL-Vir and DL-CK.

In the present work, the OPLS-DA model was established for the metabolites of LL-Avr, LL-Vir, LL-CK, DL-Avr, DL-Vir, and DL-CK to reveal the intrinsic differences within the signals (Fig. 2). The R^2^Y was 0.994, and the Q^2^ was 0.99. The results indicated that the OPLS-DA model could clearly differentiate the groups of LL-Avr, LL-Vir, LL-CK, DL-Avr, DL-Vir and DL-CK, indicating a significant difference existed between different groups (Fig. 2). The longer distance between LL-Avr and LL-Vir indicated that there were significant differences in the metabolome between these two treatment groups. Moreover, the shorter distance between DL-Avr and DL-Vir indicated that Avr and Vir could induce similar SAR response in distal leaves (Fig. 2).

### The Avr and Vir responsive metabolites in local leaves and distal leaves

**Figure 3:**
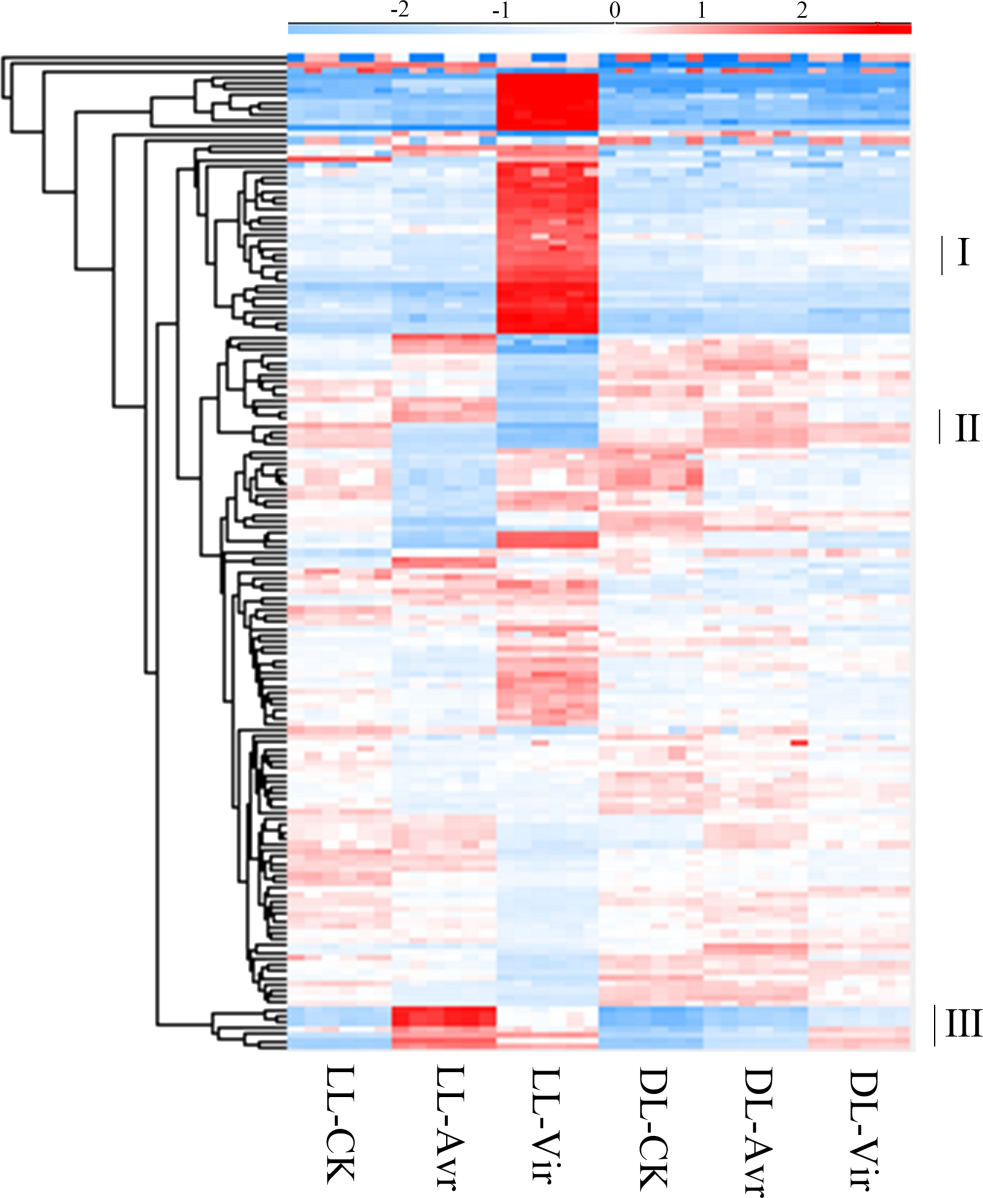
The results of the identified metabolites clustering analysis in LL-CK, LL-Avr, LL-Vir, DLCK, DL-Avr and DL-Vir groups. The raw abundance values were normalized by raw mean and log2.

In metabolomics analyses, 157 metabolites were qualitatively and quantitatively identified across six biological replicates (Table S1). The accumulation patterns of these metabolites in all the 36 samples are shown in Fig. 3. The labelled three clusters are probably related with SAR. Compared to LL-CK, metabolites in cluster III showed a higher abundance in LL-Avr and LL-Vir, including cinnamyl alcohol, adenylthiomethylpentose, aspartyl-L-proline, 3-phenylpropanoic acid, tris(hydroxymethyl)aminomethane, and sinapoylspermine. These metabolites are possibly associated with the generation of the systemic signals in local leaves. In DL-Avr and DL-Vir, the abundance of the metabolites in cluster I and cluster II was higher than that in DL-CK, including camalexin, glutamine, histidine, lysine, 5′-O-beta-D-glucosylpyridoxine, 2,4-dihydroxyheptadec-16-enyl acetate, and 2,4-dihydroxyheptadec-16-ynyl acetate. These metabolites may contribute to SAR in distal leaves.

**Figure 4:**
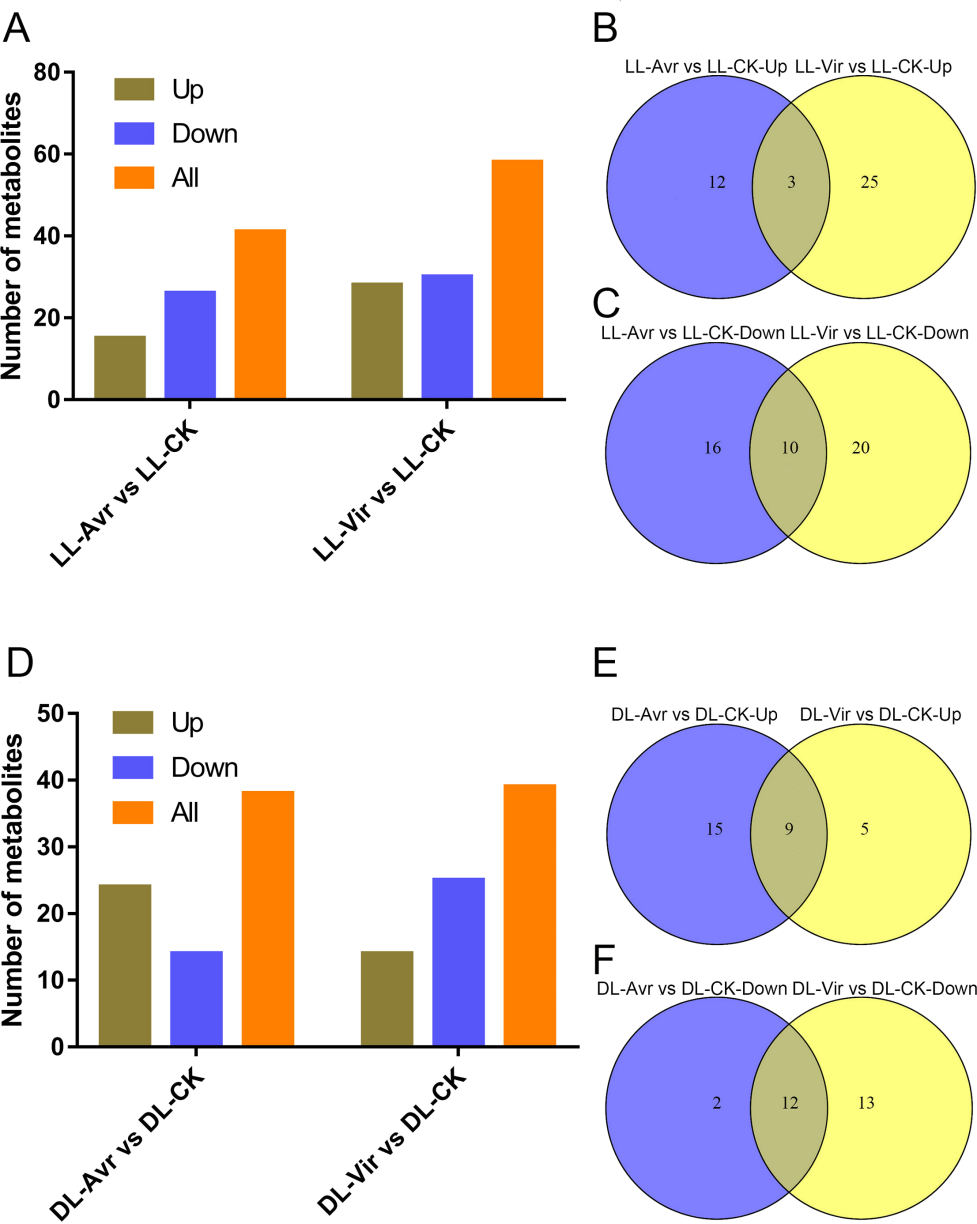
Summary of Arabidopsis metabolites response to the infection of Avr and Vir in LL and DL of locally Avr and Vir inoculated plants. (A) Number of up- or down-regulated metabolites in LL-Avr vs. LL-CK and LL-Vir vs. LL-CK. (B) Venn diagram representing up regulated metabolites in LL-Avr vs. LL-CK and LL-Vir vs. LL-CK. (C) Venn diagram representing down-regulated metabolites in LL-Avr vs LL-CK and LL-Vir vs. LL-CK. (D) Number of up- or down-regulated metabolites in DL-Avr vs. DL-CK and DL-Vir vs. DL-CK. (E) Venn diagram representing up regulated metabolites in DL-Avr vs. DLCK and DL-Vir vs. DL-CK. (F) Venn diagram representing down-regulated metabolites in DL-Avr vs. DLCK and DL-Vir vs. DL-CK.

Within the 157 identified metabolites, 41 and 58 differentially accumulated metabolites (DAMs) showed significant changes in LL-Avr vs. LL-CK and LL-Vir vs. LL-CK, respectively (fold change < 0.83 or >1.2, VIP >1, *P* < 0.05, Table S2). Compared to LL-CK, the contents of 15 DAMs are increased in LL-Avr, including 2′-hydroxy-4, 4′, 6′-trimethoxychalcone, 3-O-feruloyl-D-quinic acid, sinapinic acid, citrulline, citric acid, ketoglutaric acid, hexadecasphinganine, and the contents of 26 DAMs are decreased in LL-Avr (Fig. 4A, Table S2), including threonine, cinnamic acid, leucine, and valine (Table 1). Compared to LL-CK, 28 DAMs showed an increased accumulation level and 30 DAMs showed a decreased accumulation level in LL-Vir (Fig. 4A, Table S2). The increased DAMs include benzoic acid, valine, theanine, phenylalanine, lysine, histidine, and hexadecasphinganine. The decreased DAMs include sinapinic acid, glutamine, glutamic acid, and aspartic acid (Table 1).

Venn diagram analysis showed that 3 DAMs were commonly up-accumulated both in LL-Avr and in LL-Vir (Fig. 4B, Table S3), and 10 DAMs were commonly decreased in LL-Avr and in LL-Vir (Fig. 4C, Table S3). The interesting DAMs include hexadecasphinganine, trans-3-indoleacrylic acid, arginine, and choline (Table 1). To investigate the DAMs related with SAR in distal leaves, we then focused on the analysis of the DAMs in DL-Avr and in DL-Vir. As shown in Fig. 4D, 24 and 14 DAMs are up-accumulated in DL-Avr and DL-Vir, while 14 and 25 DAMs are decreased in DL-Avr and DL-Vir, respectively (fold change < 0.83 or > 1.2, VIP > 1, *P* < 0.05, Table S2). Among them, 9 DAMs are commonly increased (Fig. 4E, Table S4) and 12 DAMs are commonly decreased (Fig. 4F, Table S4). Numerous DAMs are interesting, such as 2′-hydroxy-4, 4′, 6′-trimethoxychalcone, glutamine, phenylalanine, pyroglutamic acid, and (9S,13S)-12-oxophytodienoic acid.

### Identification of DAMs associated with SAR

Except for transcriptional reprogramming and activation of immunity signalling pathways, defense responses can also affect the accumulation of metabolites, including organic acids, amino acids, phenolic compounds, and many defense-associated phytohormones. In the present work, the metabolite profiles were investigated to explore the DAMs associated with SAR. In LL-Avr vs. LL-CK or LL-Vir vs. LL-CK, several phenolic compounds were differentially accumulated, such as 3-O-feruloyl-D-quinic acid, sinapinic acid, coumarone, *p*-coumaric acid, and cinnamic acid (Table 1). In addition, the content of 14 amino acids and their derivatives were significantly changed, such as glutamine, leucine, valine, glutamic acid, asparagine, and aspartic acid (Table 1). Moreover, citric acid and ketoglutaric acid showed a significant up-accumulation in LL-Avr, and the content of 2-methylcitric acid was decreased in LL-Vir (Table 1). Besides, the accumulation level of many other interesting metabolites were significantly changed in local leaves, such as adenine, adenosine, hexadecasphinganine, choline, and (9S, 13S)-12-oxophytodienoic acid.

**Table 1:** List of differentially expressed metabolites (DAMs) in LL-Avr vs. LL-CK, LL-Vir vs. LL-CK, DL-Avr vs. DL-CK and DL-Vir vs. DL-CK. Vir, virulent *Psm ES4326*; Avr, avirulent *Psm avrRpm1*; CK, MgCl_2_.

**Metabolites**	**Formula**	**Fold change**			
		**LL-Vir vs. LL-CK**	**LL-Avr vs. LL-CK**	**DL-Vir vs. DL-CK**	**DL-Avr vs. DL-CK**
Phenolic compounds					
2′-Hydroxy-4,4′,6′-trimethoxychalcone	C18 H18 O5	5.2	5.1	4.7	1.8
3-O-feruloyl-D-quinic acid	C17 H20 O9	0.37	1.4	/	/
Sinapinic acid	C13 H23 N7 O9	0.23	1.6	/	1.9
Cinnamic acid	C9 H8 O2	3.6	0.5	1.7	/
P-coumaric acid	C9 H8 O3	8.4	/	1.3	1.72
Coumarone	C8 H6 O	9	/	/	1.8
octyl methoxycinnamate	C18 H26 O3	3.1	/	/	/
Benzoic acid	C7 H6 O2	5.9	/	/	/
Salicylic acid	C7 H6 O3	4.25	/	/	/
Amino acids and its derivatives					
Glutamine	C5 H10 N2 O3	0.53	/	1.35	1.72
Leucine	C6 H13 N O2	1.66	0.67	1.48	1.64
Serine	C3 H7 N O3	0.54	/	1.33	1.65
Valine	C5 H11 N O2	1.94	0.61	1.4	1.46
Theanine	C7 H14 N2 O3	2.6	/	/	1.35
Phenylalanine	C9 H11 N O2	3.7	/	1.85	1.37
Glutamic acid	C5 H9 N O4	0.5	/	/	1.25
Asparagine	C4 H8 N2 O3	0.6	/	1.22	/
Proline	C5 H9 N O2	/	0.83	0.83	/
Lysine	C6 H14 N2 O2	4.4	/	/	/
Histidine	C6 H9 N3 O2	4.7	/	/	/
Arginine	C6 H14 N4 O2	0.4	0.83	/	/
Aspartic acid	C4 H7 N O4	0.57	0.73	/	/
Threonine	C4 H9 N O3	1.51	0.69	/	/
Citrulline	C6 H13 N3 O3	0.73	1.25	/	1.57
Tiglylglycine	C7 H11 N O3	2.56	/	/	1.33
Pyroglutamic acid	C5 H7 N O3	0.53	/	1.3	1.72
Organic acids and sugars					
1,2-bis-O-sinapoyl *β*-D-glucoside	C28 H32 O14	0.24	1.68	/	1.79
2-methylcitric acid	C7 H10 O7	0.18	/	0.68	0.78
cyclo-Dopa 5-O-glucoside	C15 H19 N O9	0.2	/	0.56	/
Itaconic acid	C5 H6 O4	0.51	/	0.74	/
*α*-Lactose	C12 H22 O11	0.34	0.74	0.81	/
D-Glucose 6-phosphate	C6 H13 O9 P	0.54	/	/	/
Glycerophosphoglycerol	C6 H15 O8 P	0.3	/	0.55	/
Citric acid	C6 H8 O7	/	15.04	/	/
alpha-Ketoglutaric acid	C5 H6 O5	/	14.95	/	/
5-Hydroxy-2-furoic acid	C5 H4 O4	/	15.17	/	/
Nucleotides and its derivatives					
Adenine	C5 H5 N5	/	0.29	0.41	0.41
Adenosine	C10 H13 N5 O4	/	0.28	0.37	0.38
Adenosine 5′-monophosphate	C10 H14 N5 O7 P	/	/	0.37	0.5
uridine 5′-diphosphate	C9 H14 N2 O12 P2	0.47	/	0.78	/
Coenzymes and its derivatives					
5′-O-beta-D-Glucosylpyridoxine	C14 H21 N O8	0.49	0.65	/	/
6-Decylubiquinol	C19 H32 O4	0.06	0.29	1.72	1.75
Others					
Hexadecasphinganine	C16 H35 N O2	57.38	3.35	/	/
Choline	C5H14NO	0.65	0.63	0.72	0.55
(9S,13S)-12-Oxophytodienoic acid	C18 H28 O3	/	0.2	0.43	0.71
Ethyl docosahexaenoate	C24 H36 O2	11.3	15.2	8.4	2.19
Camalexin	C11 H8 N2 S	178.2	/	0.72	0.81

**Notes.**

/ represented this metabolite was not detected in this group.

In DL-Avr vs. DL-CK or DL-Vir vs. DL-CK, many DAMs, including organic acids, amino acids and phenolic compounds, were differentially accumulated. Except proline, the content of almost all the detected amino acids was increased, such as glutamine, leucine, serine, valine, phenylalanine, and asparagine (Table 1). Besides, the accumulation level of all the detected phenolic compounds was increased in DL-Avr or DL-Vir, including 2′-hydroxy-4, 4′, 6′-trimethoxychalcone, sinapinic acid, cinnamic acid, *p*-coumaric acid and coumarone (Table 1). These results indicated that up-accumulation of amino acids and phenolic compounds was associated with SAR.

**Figure 5:**
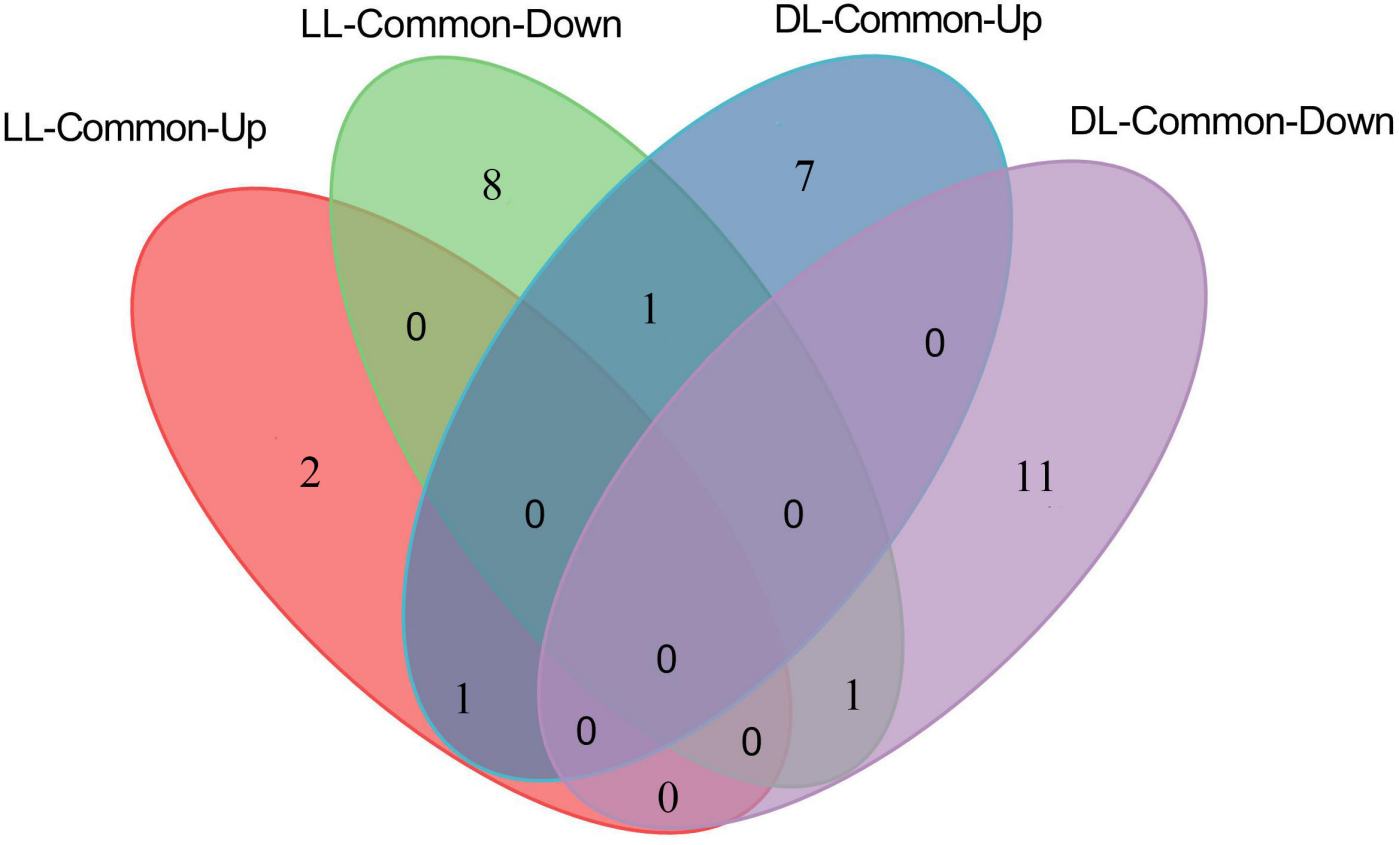
Number of up- and down-regulated metabolites commonly shared by LL-Avr vs. LL-CK and LL-Vir vs. LL-CK, DL-Avr vs. DL-CK and DL-Vir vs. DL-CK.

Since both Avr and Vir strains can induce SAR (Gruner et al., 2013), the DAMs that are consistently different in LL-Vir and LL-Avr or DL-Vir and DL-Avr are considered to be more related to SAR. Numerous DAMs showed the same accumulation tendency in LL-Vir and LL-Avr (termed as LL-common), such as 2′-hydroxy-4, 4′, 6′-trimethoxychalcone, aspartic acid, and hexadecasphinganine. In like manner, a number of DAMs displayed the same accumulation tendency in DL-Vir and DL-Avr (termed as DL-common), including coumaric acid, glutamine, leucine, serine, valine, phenylalanine, and adenine (Figs. 4B–4C, 4E and 4F, Table 1). Two DAMs showed the same accumulation trend both in LL-Common and DL-Common (Fig. 5). In detail, 2′-hydroxy-4, 4′, 6′-trimethoxychalcone was increased in LL-common and DL-common, whereas choline was decreased in LL-common and DL-common, suggesting these two DAMs were probably involved in signalling of SAR (Fig. 5, Table 1).

## Discussion

**Figure 6:**
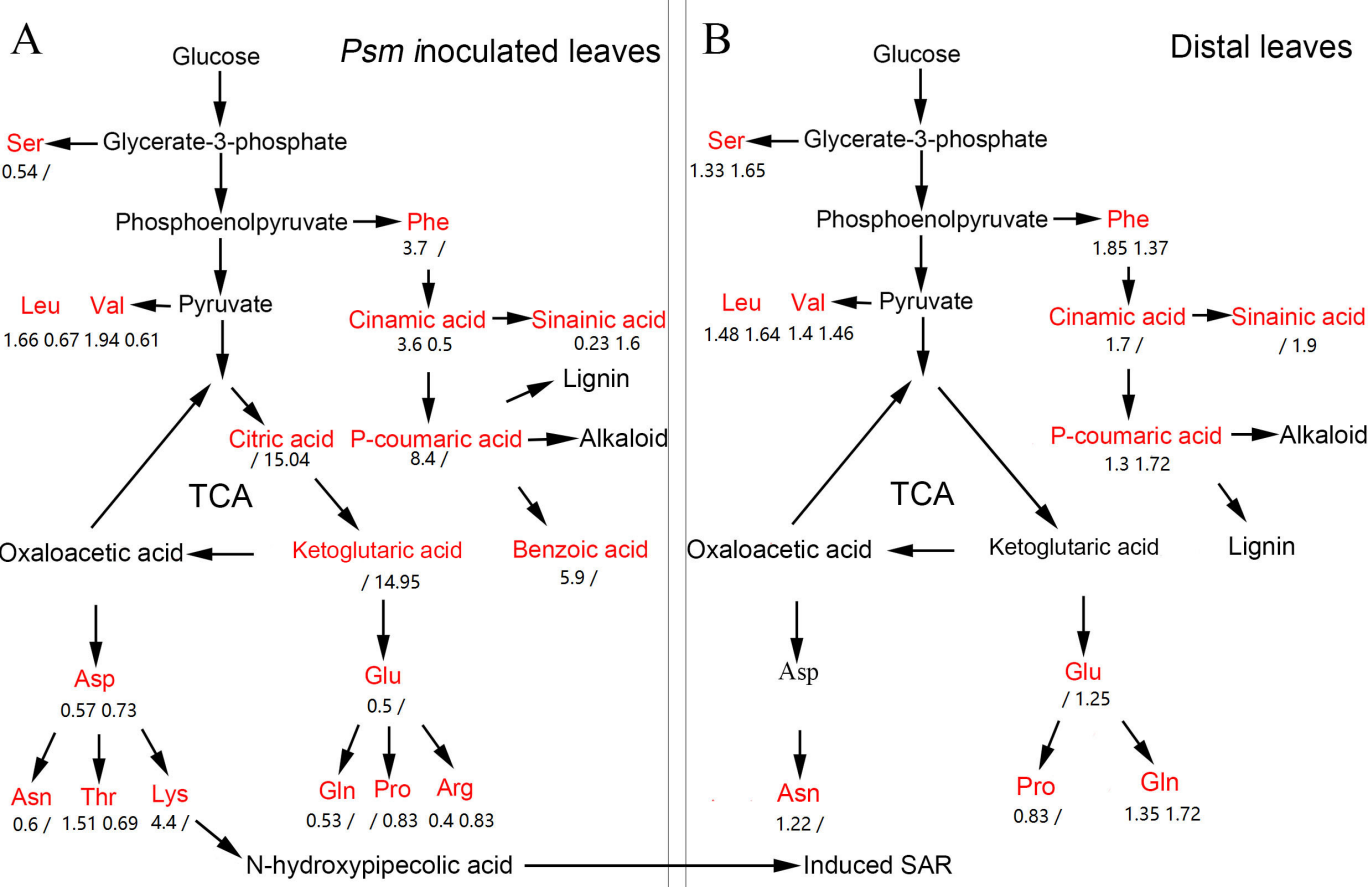
The pathway map involved in DAMs associated with phenolic compounds, amino acids, and organic acids in Psm-inoculated local leaves (A) and distal leaves of plants locally Psm-inoculated (B). Red represents differentially accumulated metabolites. Numbers represent fold change of metabolites in LL-Vir vs. LL-CK and LL-Avr vs. LL-CK, or in DL-Vir vs. DL-CK and DL-Avr vs. DL-CK, respectively. A ‘/’ represented that this metabolite was not detected.

Previous research showed that SAR could be fully established two days after induction by pathogens (Shah & Zeier, 2013). In the present work, the DAMs associated with SAR were identified, including phenolic compounds, amino acids, organic acids, sugars, nucleotides, coenzymes, and many other metabolites. The pathways related to these DAMs are shown in Fig. 6.

### Phenolic compounds

Phenolic compounds are often produced and accumulated in plant tissues exposed to biotic or abiotic stresses (Clé et al., 2008; Schmitz-Hoerner & Weissenböck, 2003). To date, a lot of phenolic compounds have been confirmed to be biologically active in resistance to pathogens, such as soluble phenylpropanoids, flavones, and phytoalexins (Bhattacharya, Sood & Citovsky, 2010). Balmer et al. (2013) reported that the accumulation level of phenolic compounds was dramatically changed in local leaves infected by *Colletotrichum graminicola* and in systemic leaves in maize. In the present work, a number of phenolic compounds showed significant changes in *Psm*-inoculated local leaves and in uninfected distal leaves (Table 1, Fig. 6), such as 2′-hydroxy-4, 4′, 6′-trimethoxychalcone, sinapinic acid, cinnamic acid, and *p*-coumaric acid.

As the precursors of flavonoids that are widely found in plants, chalcones possess a relatively simple structure and diverse pharmacological effects, including antibacterial and antifungal activities (Alcaraz et al., 2004; Boeck et al., 2005; Chiaradia et al., 2008; Mascarello et al., 2010). As a derivative of chalcone, 2′-hydroxy-4, 4′, 6′-trimethoxychalcone can inhibit the growth of fungal pathogens (Costa et al., 2016). As a critical intermediate in the synthesis of lignin and alkaloids, *p*-coumaric acid plays an important role in plant defense responses. Previous research showed that *p*-coumaric acid could repress *T3SS* of *Dickeya dadantii* through the HrpX/Y two-component system (Li et al., 2009). In this study, 2′-hydroxy-4, 4′, 6′-trimethoxychalcone and *p*-coumaric acid were up-accumulated both in infected local leaves and in uninfected distal leaves, suggesting these two metabolites are related to SAR.

Sinapinic acid, an intermediate in phenylpropanoids metabolism, is involved in synthesis of many secondary metabolites in plants, including SA, lignin, and sinapoylglucose. In Arabidopsis, sinapoylglucose could specifically inhibit the growth of fungal pathogens, and a mutant of a sinapinic acid synthesis gene exhibited enhanced susceptibility to *Verticillium longisporum* (König et al., 2014). Cinnamic acid (CA) is an organic acid first isolated from cinnamon bark. The antimicrobial activities of CA and its derivatives have been confirmed in *in vitro* experiments (Sadeghi et al., 2013). CA can also damage the integrity of the plasma membrane and elevate the intracellular level of reactive oxygen species in *Botrytis cinerea*, indicating reactive oxygen species appeared to be responsible for the inhibitory effects of CA on the growth of fungal pathogens (Zhang et al., 2015). In the present work, the accumulation level of sinapinic acid and cinnamic acid were significantly increased in distal leaves of locally *Psm*-inoculated plants (Table 1, Fig. 6), indicating these two metabolites may play an important role in SAR.

### Amino acids and the derivatives

It has been reported that the change of amino acid content in maize was related to the establishment of SAR (Balmer et al., 2013). In the present work, the accumulation levels of 14 amino acids and 3 amino acid derivatives were significantly changed in *Psm*-inoculated local leaves and in uninoculated distal leaves. Compared to MgCl_2_-infiltrated leaves, the contents of serine, aspartic acid, asparagine, glutamic acid, glutamine, arginine, and proline were decreased in LL-Avr and LL-Vir, whereas the contents of phenylalanine and lysine were increased. Except for proline, all the detected amino acids were up-accumulated in DL-Avr and DL-Vir, including serine, phenylalanine, leucine, valine, asparagine, glutamic acid, and glutamine (Table 1, Fig. 6).

Previous research showed that a number of plant pathogens have evolved to utilize the amino acids that are present in their hosts as carbon and nitrogen sources. For instance, the pathogenic *P. syringae pv. tomato* has been found to be nutritionally specialized to catabolize the abundant amino acids in tomato apoplast, including glutamine, glutamate, and aspartate (Rico & Preston, 2008; Seifi et al., 2013). The decrease of most amino acids in inoculated local leaves indicated that Arabidopsis is likely to cope with the infection of *P. syringae* by down-regulation of the content of some amino acids, such as glutamine, glutamate, and aspartate.

As an Asp-derived amino acid, lysine plays an important role in plant resistance to biotic stress. Loss-of-function of *AGD2-LIKE DEFENSE RESPONSE PROTEIN1* (*ALD1*), a gene involved in lysine catabolism, resulted in reduction of the SA content and attenuation of the plant resistance to *P. syringae* (Song et al., 2004; Song, Lu & Greenberg, 2004; Hudson et al., 2006). N-hydroxypipecolic acid, a product of lysine catabolism, is an immune signal which can amplify the defense responses in plants, and acts as a critical regulator in local resistance, priming of systemic immunity and SAR (Zeier, 2013). In the present work, the content of aspartic acid was decreased in *Psm*-inoculated local leaves (Table 1, Fig. 6), further confirming that lysine is a strong feedback suppressor of the Asp-pathway (Yang & Ludewig, 2014),

Phenylalanine is the main precursor of many phenolic compounds, and plays important roles in plant resistance to biotic stress, such as protecting the plant tissue from infection and inhibiting the spore germination and the hyphal growth of the fungal pathogens (Tzin & Galili, 2010; Vogt, 2010; Maeda & Dudareva, 2012; Tohge et al., 2013). Consistent with this, phenylalanine and most detected phenolic compounds were up-accumulated in *Psm*-inoculated local leaves and in distal uninfected leaves in the present work (Table 1, Fig. 6), indicating the metabolic pathway of phenylalanine might be associated with the establishment of SAR.

Glutamine is a major amino donor for the synthesis of nucleotides and amino acids. Exogenous application of glutamine could rapidly induce the key transcriptional factor genes involved in stress responses (Kan et al., 2015). In the present work, glutamine was up-accumulated in distal leaves of the locally *Psm*-inoculated plants, revealing a relationship with the activation of the stress-related genes. In summary, our data showed that amino acids played important roles in SAR in Arabidopsis.

### Organic acids

The accumulation level of 6 organic acids were significantly changed in local *Psm*-inoculated leaves or distal uninfected leaves. Two intermediates of the TCA cycle, citric acid and ketoglutaric acid, were up-accumulated in LL-Avr (Table 1, Fig. 6). The TCA cycle is a central metabolic pathway for aerobic processes, and is responsible for energy production from fatty acid, carbohydrate, and amino acids (Fernie, Carrari & Sweetlove, 2004). It has been shown that the defense induction is a cost-intensive process, and almost all of the inducible resistances are known to be highly energy-demanding and heavily rely on the intermediates generated by the TCA cycle (Berger, Sinha & Roitsch, 2007). The up-accumulation of citric acid and ketoglutaric acid indicated that the TCA cycle could be enhanced by Avr inoculation in local leaves, which could provide more energy and more intermediates for resistance to pathogens.

### Other DAMs associated with SAR

Besides phenolic compounds, amino acids, and organic acids, many other DAMs were identified as potential candidates associated with SAR, such as hexadecasphinganine, choline, and (9S,13S)-12-oxophytodienoic acid. Sphingolipids, the primary lipid components of eukaryotic membranes, play important roles in regulation of signal transduction, programmed cell death, vesicular trafficking, autophagy, and alternative splicing (Sentelle et al., 2012; Young, Kester & Wang, 2013). Mutants of *AtACER*, which encodes a ceramidase involved in hydrolyzation of sphingolipids into sphingosine and fatty acids, exhibited susceptibility to *P. syringae* (Wu et al., 2015). The data of the present work showed that hexadecasphinganine, a sphingolipid, was up-accumulated in *Psm*-inoculated local leaves (Table 1). It suggests that hexadecasphinganine may contribute to the basic resistance and the establishment of SAR.

As an important phytohormone, JA (jasmonic acid) mediates the responses against necrotrophic pathogens, and acts as a signalling molecule to facilitate the interaction between the plants and the root-associated beneficial microorganisms (Pieterse et al., 2009). Different from JA, SA plays a key role in plant immunity against biotrophic pathogens (Betsuyaku et al., 2018). It is well known that JA and SA can antagonistically regulate many processes of plant resistances to biotic stress (Betsuyaku et al., 2018). Consistently, the (9S,13S)-12-oxophytodienoic acid, a precursor of JA, was decreased in LL-Avr, DL-Vir and DL-Avr, suggesting suppression of the JA-mediated pathways contributes to the establishment of SAR.

## Conclusions

In the present work, we explored the differentially accumulated metabolites by UPLC-MS/MS at the time when SAR was just fully established. The results of this study provide a new viewpoint that will assist to better understand the complex molecular and cellular events occur during the establishment of SAR. A number of metabolites were identified as potential components associated with SAR. The significant changes of phenolic compounds, amino acids, nucleotides and nucleotide derivatives, organic acids, and other metabolites in *Psm*-inoculated local leaves and distal leaves suggest that these DAMs may play important roles in SAR. The up-accumulation of phenolic compounds in *Psm*-inoculated local leaves and distal leaves illustrates that these metabolites could be used as key intermediates for the synthesis of various secondary metabolites contributing to basal defense and systemic immunity. Furthermore, the contents of multiple amino acids are increased both in DL-Avr and DL-Vir, including leucine, serine, valine and phenylalanine, suggesting these amino acids may play a crucial role in establishment of SAR. In addition, the changed contents of various metabolites in *Psm*-inoculated local leaves and distal leaves indicate that plants can reallocate a portion of the physiological activities during the establishment of the basic resistance and the SAR to cope with the imminent infection of the pathogens.

##  Supplemental Information

10.7717/peerj.10047/supp-1 Table S1 List of total metabolites identified in LL and DL

10.7717/peerj.10047/supp-2 Table S2 List of differentially accumulated metabolites in LL-Avr vs LL-CK, LL-Vir vs LL-CK, DL-Avr vs DL-CK and DL-Vir vs DL-CK

10.7717/peerj.10047/supp-3 Table S3 List of differentially accumulated metabolites shared by LL-Avr vs LL-CK and LL-Vir vs LL-CK

10.7717/peerj.10047/supp-4 Table S4 List of differentially accumulated metabolites shared by DL-Avr vs DL-CK and DL-Vir vs DL-CK

10.7717/peerj.10047/supp-5 Table S5 The work flow and parameters of Compound Discoverer 3.0

10.7717/peerj.10047/supp-6 Table S6 Raw data of bacteria number in Fig. 1C

10.7717/peerj.10047/supp-7 Table S7 The raw data of qRT-PCR

## Additional Information and Declarations

### Data Availability

The following information was supplied regarding data availability: The mass spectrometry metabolomics data are available at EMBL-EBI MetaboLights database (https://www.ebi.ac.uk/metabolights/MTBLS1550) and raw measurements are available in the Supplementary Files.

## References

[ref-1] Ahmad S, Veyrat N, Gordon-Weeks R, Zhang Y, Martin J, Smart L, Glauser G, Erb M, Flors V, Frey M, Ton J (2011). Benzoxazinoid metabolites regulate innate immunity against aphids and fungi in maize. Plant Physiology.

[ref-2] Alcaraz MJ, Vicente AM, Araico A, Dominguez JN, Terencio MC, Ferrándiz ML (2004). Role of nuclear factor- *κ*B and heme oxygenase-1 in the mechanism of action of an anti-inflammatory chalcone derivative in RAW 264.7 cells. British Journal of Pharmacology.

[ref-3] Balmer D, De Papajewski DV, Planchamp C, Glauser G, Mauch-Mani B (2013). Induced resistance in maize is based on organ-specific defence responses. Plant Journal.

[ref-4] Berger S, Sinha AK, Roitsch T (2007). Plant physiology meets phytopathology: plant primary metabolism and plant–pathogen interactions. Journal of Experimental Botany.

[ref-5] Betsuyaku S, Katou S, Takebayashi Y, Sakakibara H, Nomura N, Fukuda H (2018). Salicylic acid and jasmonic acid pathways are activated in spatially different domains around the infection site during effector-triggered immunity in *Arabidopsis thaliana*. Plant and Cell Physiology.

[ref-6] Bhattacharjee S, Halane MK, Kim SH, Gassmann W (2011). Pathogen effectors target Arabidopsis EDS1 and alter its interactions with immune regulators. Science.

[ref-7] Bhattacharya A, Sood P, Citovsky V (2010). The roles of plant phenolics in defence and communication during *Agrobacterium* and *Rhizobium* infection. Molecular Plant Pathology.

[ref-8] Boeck P, Leal PC, Yunes RA, Filho VC, López S, Sortino M, Escalante A, Furlán RLE, Zacchino S (2005). Antifungal activity and studies on mode of action of novel xanthoxyline-derived chalcones. Archiv der Pharmazie.

[ref-9] Cameron RK, Dixon RA, Lamb CJ (1994). Biologically induced systemic acquired resistance in *Arabidopsis thaliana*. Plant Journal.

[ref-10] Cao J, Li M, Chen J, Liu P, Li Z (2016). Effects of Me JA on Arabidopsis metabolome under endogenous JA deficiency. Scientific Reports.

[ref-11] Carella P, Merl-Pham J, Wilson DC, Dey S, Hauck SM, Vlot AC, Cameron RK (2016). Comparative proteomics analysis of phloem exudates collected during the induction of systemic acquired resistance. Plant Physiology.

[ref-12] Chanda B, Xia Y, Mandal MK, Yu K, Sekine KT, Gao QM, Selote D, Hu Y, Stromberg A, Navarre D, Kachroo A, Kachroo P (2011). Glycerol-3-phosphate is a critical mobile inducer of systemic immunity in plants. Nature Genetics.

[ref-13] Chen YC, Holmes EC, Rajniak J, Kim JG, Tang S, Fischer CR, Mudgett MB, Sattely ES (2018). N-hydroxy-pipecolic acid is a mobile metabolite that induces systemic disease resistance in Arabidopsis. Proceedings of the National Academy of Sciences of the United States of America.

[ref-14] Chiaradia LD, Santos Rdos, Vitor CE, Vieira AA, Leal PC, Nunes RJ, Calixto JB, Yunes RA (2008). Synthesis and pharmacological activity of chalcones derived from 2, 4, 6-trimethoxyacetophenone in RAW 264.7 cells stimulated by LPS: quantitative structure-acetivity relationships. Bioorganic & Medicinal Chemistry.

[ref-15] Clé C, Hill LM, Niggeweg R, Martin CR, Guisez Y, Prinsen E, Jansen MAK (2008). Modulation of chlorogenic acid biosynthesis in *Solanum lycopersicum*; consequences for phenolic accumulation and UV-tolerance. Phytochemistry.

[ref-16] Costa GM, Endo EH, Cortez DAG, Nakamura TU, Nakamura CV, Filho BPDias (2016). Antimicrobial effects of *Piper hispidum* extract, fractions and chalcones against *Candida albicans* and *Staphylococcus aureus*. Journal de Mycologie Medicale.

[ref-17] De Vos RCH, Moco S, Lommen A, Keurentjes JJB, Bino RJ, Hall RD (2007). Untargeted large-scale plant metabolomics using liquid chromatography coupled to mass spectrometry. Nature Protocols.

[ref-18] Dettmer K, Aronov PA, Hammock BD (2007). Mass spectrometry-based metabolomics. Mass Spectrometry Reviews.

[ref-19] Farahbakhsh F, Hamzehzarghani H, Massah A, Tortosa M, Yassaie M, Rodriguez VM (2019). Comparative metabolomics of temperature sensitive resistance to wheat streak mosaic virus (WSMV) in resistant and susceptible wheat cultivars. Journal of Plant Physiology.

[ref-20] Fernie AR, Carrari F, Sweetlove LJ (2004). Respiratory metabolism: glycolysis, the TCA cycle and mitochondrial electron transport. Current Opinion in Plant Biology.

[ref-21] Grata E, Boccard J, Guillarme D, Glauser G, Carrupt PA, Farmer EE, Wolfender JL, Rudaz S (2008). UPLC-TOF-MS for plant metabolomics: a sequential approach for wound marker analysis in *Arabidopsis thaliana*. Journal of Chromatography B-analytical Technologies in The Biomedical and Life Sciences.

[ref-22] Gruner K, Griebel T, Návarová H, Attaran E, Zeier J (2013). Reprogramming of plants during systemic acquired resistance. Frontier in Plant Science.

[ref-23] Hartmann M, Zeier T, Bernsdorff F, Reichel-Deland V, Kim D, Hohmann M, Scholten N, Schuck S, Bräutigam A, Hölzel T, Ganter C, Zeier J (2018). Flavin monooxygenase-generated N-hydroxypipecolic acid is a critical element of plant systemic immunity. Cell.

[ref-24] Hudson AO, Singh BK, Leustek T, Gilvarg C (2006). An LL-diaminopimelate aminotransferase defines a novel variant of the lysine biosynthesis pathway in plants. Plant Physiology.

[ref-25] Johansson ON, Nilsson AK, Gustavsson MB, Backhaus T, Andersson MX, Ellerström M (2015). A quick and robust method for quantification of the hypersensitive response in plants. PeerJ.

[ref-26] Jung HW, Tschaplinski TJ, Wang L, Glazebrook J, Greenberg JT (2009). Priming in systemic plant immunity. Science.

[ref-27] Kan CC, Chung TY, Juo YA, Hsieh MH (2015). Glutamine rapidly induces the expression of key transcription factor genes involved in nitrogen and stress responses in rice roots. BMC Genomics.

[ref-28] Kang K, Yue L, Xia X, Liu K, Zhang W (2019). Comparative metabolomics analysis of different resistant rice varieties in response to the brown planthopper *Nilaparvata lugens* Hemiptera: Delphacidae. Metabolomics.

[ref-29] König S, Feussner K, Kaever A, Landesfeind M, Thurow C, Karlovsky P, Gatz C, Polle A, Feussner I (2014). Soluble phenylpropanoids are involved in the defense response of Arabidopsis against *Verticillium longisporum*. New phytologist.

[ref-30] Kunze G, Zipfel C, Robatzek S, Niehaus K, Boller T, Felix G (2004). The N terminus of bacterial elongation factor Tu elicits innate immunity in Arabidopsis plants. The Plant Cell.

[ref-31] Li Y, Peng Q, Selimi D, Wang Q, Charkowski AO, Chen X, Yang CH (2009). The plant phenolic compound *p*-coumaric acid represses gene expression in the *Dickeya dadantii* type III secretion system. Applied and Environmental Microbiology.

[ref-32] Liu YY, Yang ZX, Ma L, Wen X, Ji H, Li K (2019). ^1^H-NMR spectroscopy identifies potential biomarkers in serum metabolomic signatures for early stage esophageal squamous cell carcinoma. PeerJ.

[ref-33] Maeda H, Dudareva N (2012). The shikimate pathway and aromatic amino acid biosynthesis in plants. Annual Review of Plant Biology.

[ref-34] Mascarello A, Chiaradia LD, Vernal J, Villarino A, Guido RVC, Perizzolo P, Poirier V, Wong D, Martins PGA, Nunes RJ, Yunes RA, Andricopulo AD, Av-Gay Y, Terenzi H (2010). Inhibition of *Mycobacterium tuberculosis* tyrosine phosphatase PtpA by synthetic chalcones: kinetics, molecular modeling, toxicity and effect on growth. Bioorganic & Medicinal Chemistry.

[ref-35] Návarová H, Bernsdorff F, Döring AC, Zeier J (2012). Pipecolic acid, an endogenous mediator of defense amplification and priming, is a critical regulator of inducible plant immunity. The Plant Cell.

[ref-36] Nürnberger T, Brunner F (2002). Innate immunity in plants and animals: emerging parallels between the recognition of general elicitors and pathogen-associated molecular patterns. Current Opinion in Plant Biology.

[ref-37] Park SW, Kaimoyo E, Kumar D, Mosher S, Klessig DF (2007). Methyl salicylate is a critical mobile signal for plant systemic acquired resistance. Science.

[ref-38] Pieterse CM, Leon-Reyes A, Van der Ent S, Van Wees SC (2009). Networking by small-molecule hormones in plant immunity. Nature Chemistry Biology.

[ref-39] Rico A, Preston GM (2008). Pseudomonas syringae pv. tomato DC3000 uses constitutive and apoplast-induced nutrient assimilation pathways to catabolize nutrients that are abundant in the tomato apoplast. Molecular Plant-Microbe Interactions.

[ref-40] Sadeghi M, Zolfaghari B, Senatore M, Lanzotti V (2013). Antifungal cinnamic acid derivatives from Persian leek (*Allium ampeloprasum* Subsp. Persicum). Phytochemistry Letters.

[ref-41] Scandiani MM, Luque AG, Razori MV, Casalini LC, Aoki T, O’Donnell K, Cervigni GDL, Spampinato CP (2015). Metabolic profiles of soybean roots during early stages of *Fusarium tucumaniae* infection. Journal of Experimental Botany.

[ref-42] Schauer N, Semel Y, Roessner U, Gur A, Balbo I, Carrari F, Pleban T, Perez-Melis A, Bruedigam C, Kopka J, Willmitzer L, Zamir D, Fernie AR (2006). Comprehensive metabolic profiling and phenotyping of interspecific introgression lines for tomato improvement. Nature Biotechnology.

[ref-43] Schmitz-Hoerner R, Weissenböck G (2003). Contribution of phenolic compounds to the UV-B screening capacity of developing barley primary leaves in relation to DNA damage and repair under elevated UV-B levels. Phytochemistry.

[ref-44] Schwachtje J, Fischer A, Erban A, Kopka J (2018). Primed primary metabolism in systemic leaves: a functional systems analysis. Scientific Reports.

[ref-45] Seifi HS, Bockhaven JV, Angenon G, Höfte M (2013). Glutamate metabolism in plant disease and defense: friend or foe?. Molecular Plant-Microbe Interactions.

[ref-46] Sentelle RD, Senkal CE, Jiang W, Ponnusamy S, Gencer S, Selvam SP, Ramshesh VK, Peterson YK, Lemasters JJ, Szulc ZM, Bielawski J, Ogretmen B (2012). Ceramide targets autophagosomes to mitochondria and induces lethal mitophagy. Nature Chemical Biology.

[ref-47] Shah J, Zeier J (2013). Long-distance communication and signal amplification in systemic acquired resistance. Frontier in Plant Science.

[ref-48] Song JT, Lu H, Greenberg JT (2004). Divergent roles in *Arabidopsis thaliana* development and defense of two homologous genes, ABERRANT GROWTH AND DEATH2 and *AGD2-LIKE DEFENSE RESPONSE PROTEIN1*, encoding novel aminotransferases. The Plant Cell.

[ref-49] Song JT, Lu H, McDowell JM, Greenberg JT (2004). A key role for ALD1 in activation of local and systemic defenses in Arabidopsis. Plant Journal.

[ref-50] Spoel SH, Dong X (2012). How do plants achieve immunity? Defence without specialized immune cells. Nature Reviews Immunology.

[ref-51] Tohge T, Watanabe M, Hoefgen R, Fernie AR (2013). Shikimate and phenylalanine biosynthesis in the green lineage. Frontier in Plant Science.

[ref-52] Tzin V, Galili G (2010). The biosynthetic pathways for shikimate and aromatic amino acids in *Arabidopsis thaliana*. The Arabidopsis Book.

[ref-53] Vogt T (2010). Phenylpropanoid biosynthesis. Molecular Plant.

[ref-54] Wang XY, Li DZ, Li Q, Ma YQ, Yao JW, Huang X, Xu ZQ (2016). Metabolomic analysis reveals the relationship between *AZI1* and sugar signaling in systemic acquired resistance of Arabidopsis. Plant Physiology and Biochemistry.

[ref-55] Ward JL, Forcat S, Beckmann M, Bennett M, Miller SJ, Baker JM, Hawkins ND, Vermeer CP, Lu C, Lin W, Truman WM, Beale MH, Draper J, Mansfield JW, Grant M (2010). The metabolic transition during disease following infection of *Arabidopsis thaliana* by *Pseudomonas syringae* pv. tomato. Plant Journal.

[ref-56] Wu JX, Li J, Liu Z, Yin J, Chang ZY, Rong C, Wu JL, Bi FC, Yao N (2015). The Arabidopsis ceramidase AtACER functions in disease resistance and salt tolerance. Plant Journal.

[ref-57] Wu S, Tohge T, Cuadros-Inostroza Á, Tong H, Tenenboim H, Kooke R, Méret M, Keurentjes JB, Nikoloski Z, Fernie AR, Willmitzer L, Brotman Y (2018). Mapping the Arabidopsis metabolic landscape by untargeted metabolomics at different environmental conditions. Molecular Plant.

[ref-58] Yang H, Ludewig U (2014). Lysine catabolism, amino acid transport, and systemic acquired resistance: what is the link?. Plant Signaling & Behavior.

[ref-59] Young MM, Kester M, Wang HG (2013). Sphingolipids: regulators of crosstalk between apoptosis and autophagy. Journal of Lipid Research.

[ref-60] Zeier J (2013). New insights into the regulation of plant immunity by amino acid metabolic pathways. Plant Cell and Environment.

[ref-61] Zhang Z, Qin G, Li B, Tian S (2015). Effect of cinnamic acid for controlling gray mold on table grape and its possible mechanisms of action. Current Microbiology.

[ref-62] Zhang W, Zhao F, Jiang L, Chen C, Wu L, Liu Z (2018). Different pathogen defense strategies in Arabidopsis: more than pathogen recognition. Cell.

